# AI to the rescue of voltage imaging

**DOI:** 10.1016/j.crmeth.2023.100505

**Published:** 2023-06-16

**Authors:** Jérôme A. Lecoq, Kaspar Podgorski, Benjamin F. Grewe

**Affiliations:** 1 Allen Institute, MindScope program, Seattle, USA; 2 Allen Institute, Neural Dynamics program, Seattle, USA; 3 Institute of Neuroinformatics, University of Zurich and ETH Zurich, Zurich, Switzerland; 4 ETH AI Center, ETH Zurich, Zurich, Switzerland

## Abstract

In a recent issue of *Nature Methods*, Platisa et al. present an approach for long-term, *in vivo* population voltage imaging with single spike resolution across a local population of 100 neurons.^1^ Key to this step forward was the combination of a customized high-speed two-photon microscope with an optimized, positive-going, genetically encoded voltage indicator and a tailored machine learning denoising algorithm.

## Main text

To date, the two most common technologies for monitoring neuronal population activity in the intact brain are electrophysiological recordings and calcium imaging. Multi-electrode recording based on silicon probes, e.g., NeuroPixels, enables monitoring of brain-wide neuronal spiking activity.^2^ NeuroPixels allow high-speed recordings from large numbers of neurons through 4 mm or more of deep brain tissue. Depending on the location and animal species, a single probe can record between 200 and 400 neurons simultaneously along its shaft.^2^ However, electrophysiological recordings generally lack the ability to define the genetic identity of individual cells. Also, longitudinal tracking of neural signals over weeks and months is challenging, and in some cases, the geometry of the silicon shaft limits recordings to a fraction of the population in the brain area of interest.

In contrast, *in vivo* two-photon calcium imaging provides dense recordings in deep brain areas that are optically accessible,^3^ in some cases recording the majority of neurons in a given volume. Multi-photon imaging techniques also allow the linking of neuronal activity to cellular morphology, subcellular compartments, or the genetic identities of neurons. Moreover, genetic calcium indicators have vastly improved over the last decade in terms of their sensitivity and kinetics, thanks to iterative protein engineering by groups such as the GENIE Project at Janelia Research Campus. For example, GENIE’s recently released jGCaMP8f variant exhibits a 37% ΔF/F change upon a single spike in cultured neurons and an 87 ms half-decay time.

However, calcium ions are only indirectly related to action potential firing,^4^ as several other factors modulate intracellular calcium. This problem can be overcome using indicators that directly report membrane voltage. Utilizing such voltage indicators in combination with two-photon imaging offers the same genetic specificity and dense population monitoring capabilities and promises more precise timing and counting of spikes. In addition, voltage imaging can detect subthreshold voltage signals (e.g., excitatory postsynaptic potentials [EPSPs], neural oscillations) in somatic and dendritic compartments. Two-photon voltage imaging of large neuronal populations thus represents a major direction forward in modern systems neuroscience as it could enable a more comprehensive understanding of neuronal dynamics.

However, both *in vivo* calcium and voltage imaging using two-photon microscopy are facing a fundamental challenge: to monitor neuronal activity, one must record each individual image pixel with a sufficiently high signal to noise ratio (SNR) and optical resolution. Naturally, voltage imaging of spiking signals requires frame rates of several hundreds to thousands of Hz. As a result, the photon flux often falls below a single fluorescence photon per image pixel. In such low-light conditions individual frames are highly affected by photon shot noise and it is often impossible to assign functional signals to small structures.

Although structures can be visualized by averaging frames, this reduces the sampling rate to the point one can no longer resolve neuronal spiking. Raising excitation power increases the sample brightness, but also rapidly increases photobleaching and damage.^5^ More practical approaches are to develop brighter voltage indicators or to improve microscope performance. Recent work on high-speed two-photon microscopy^6^^,^^7^ or voltage indicators^7^ has demonstrated such improvements.

Another orthogonal approach capitalizes on the information shared among nearby pixels in space and time to better extract information from shot-noise-limited recordings. In multi-photon imaging, nearby pixels within the same frame or temporally adjacent frames offer extra bits of information about the state of a given cell. Although existing methods for extracting signals from fluorescence recordings take multiple pixels and time points into account, these methods are composed of sequential steps that are each affected by noise. Computer vision methods can learn the complex spatiotemporal structure of a given recording to directly estimate the brightness of each pixel from its context in the raw recording. Key to these methods, like DeepInterpolation,^8^ is the recognition that shot-noise dominated frames provide a noisy yet unbiased estimate of ground truth signals. Therefore, these frames can be used to train deep neuronal networks to reconstruct denoised versions of multi-photon movies directly from the raw data. The reconstructed movies can then be used by traditional segmentation algorithms to extract signals.

The recent study, Platisa, et al. is special in that, for the first time, it effectively integrates several of the above approaches to achieve *in vivo* two-photon voltage imaging across a large population of individual neurons over elongated periods of time.^1^ First, Platisa et al. modified ASAP3, an existing voltage sensor,^7^ to report voltage changes with positive fluorescence changes instead of negative ones. Although the sign of signals has little effect on their detectability, positive-going voltage indicators exhibit dimmer fluorescence at rest, which can reduce photobleaching^9^ and phototoxicity. Further, a dimmer baseline fluorescence reduces shot noise due to optical crosstalk from other labeled cells. The newly presented voltage indicator, SpikeyGi2, brightens by approximately 13% ΔF/F per detected putative action potential. Voltage indicators are under rapid development and precise *in vivo* validation is challenging, but these recordings compare favorably to ASAP3. Platisa et al. measured a −7.9% ΔF/F change per event with ASAP3 in the same *in vivo* condition. This result is on par with a −9% ΔF/F change reported by the original ASAP3 publication for *in vivo* events.

**Figure 1:**
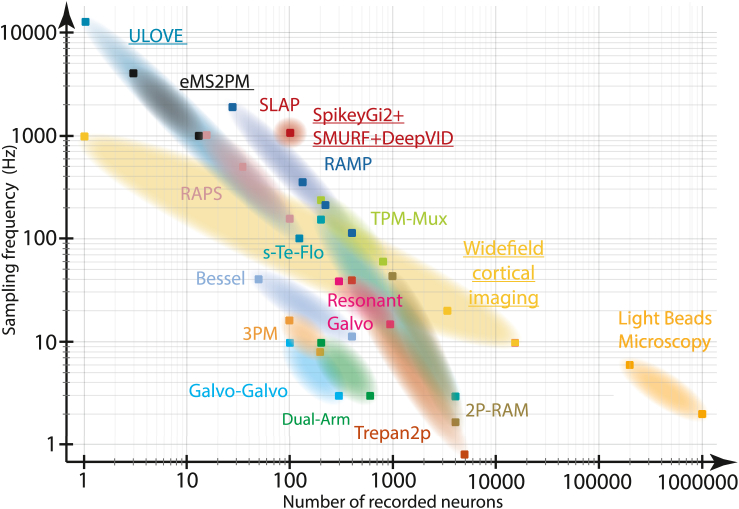
Comparison of *in vivo* imaging techniques in terms of number of neurons and recording speed onto a 2D space (number of imaged neurons vs. sampling rate) Square markers represent published data. Clouds represent hypothetical interpolation between the reported data points. Underlined labels refer to techniques that were specifically used for voltage imaging. Adapted from Lecoq et al.^10^

Second, Platisa et al. customized a two-photon imaging scheme based on spatiotemporal multiplexing^10^ to reach a 1 kHz frame rate over a 400 × 400 μm field of view (FOV). Their FOV was divided into eight bands scanned by four pairs of multiplexed beams that in some cases contained >100 neurons. The reported optical resolution of 0.9 μm (lateral) and 4.4 μm (axial) is sufficient to resolve individual neurons and even subcellular structures. Though in principle the ULoVE imaging approach could record from a larger population of neurons, especially if combined with self-supervised denoising, the original publication of ULoVE only reported a few neurons recorded at a given time *in vivo.*^7^ The combination of techniques introduced by Platisa et al. therefore provides a significant increase in the number of simultaneously recorded neurons with voltage imaging (see Figure 1 for a comparison of neuronal yield with other existing techniques).

A notable limitation of the presented approach is the remaining crosstalk of up to 10% between imaging channels. This is a known challenge associated with increasingly popular multiplexing techniques that can have subtle consequences on population analyses.^11^ In this particular instantiation, temporal crosstalk between consecutive pixels in time contributed less than 5% to the overall crosstalk of 10% that was dominated by light scattering between nearby pixels in space. For future iterations of voltage imaging systems, we anticipate the development of methods to minimize this issue, for example by utilizing computational unmixing of temporally multiplexed recordings.

Last, Platisa, et al. adapted an image denoising method like DeepInterpolation^8^ for voltage imaging to computationally reconstruct pixel intensity values in low-light conditions. Their denoising method, DeepVID, reconstructs a center frame from preceding and successive frames. In previous iterations of self-supervised denoising, the center frame served as a training target but was excluded from the input. Their modified algorithm introduced a dropout layer to utilize this central frame without causing excessive over-fitting. This center frame is particularly important for voltage imaging, as it facilitates the reconstruction of fast, brief events such as fluorescence spikes generated from action potentials.

To date, most large-scale voltage imaging approaches operate at the edge of our technological capabilities. In line with Platisa et al., we project that the next set of technological advances in neuroscience will be generated by integrating several cutting-edge technologies. In particular, we see the incorporation of deep learning methods playing a crucial role, whether it is to automatically detect and extract neural signals or to identify more potent sensors. Moreover, modern neuroscience is increasingly built upon foundational datasets acquired using such complex and integrated instruments. These datasets, similar to brain-wide cell type characterization or large-scale connectomics using electron microscopy, have been greatly utilized by the neuroscience community to create a complex ecosystem of interdependent publications. Acquiring such foundational datasets only became possible due to meticulous engineering integration and scaling. We expect these integrative efforts to continue,^10^ with more open-source foundational datasets to be accessible in the near future.

A significant repercussion of a comprehensive technological integration is that imaging devices are increasingly becoming more intricate, making them difficult to construct, transport, and reproduce. Consequently, staying abreast of numerous technological advancements is becoming an ever-greater challenge for individual neuroscience laboratories. Analogous to modern astronomical observatories that are built through national efforts, we thus see the emergence of an ecosystem of brain observatories^12^ as beneficial to the neuroscience community. Such an ecosystem would enable the rapid transformation of newly emerging, cutting-edge imaging tools into practical neuroscience research systems. By eliminating technological barriers that individual neuroscience labs face, such an ecosystem could give widespread access to a multitude of these novel imaging tools. For the future, we hope for more of such technological ecosystems to emerge, which we consider crucial for the field to advance our understanding of complex brain interactions that arise from the concerted activity of millions of neurons.
